# Assessment of the Transmission Dynamics of *Clostridioides difficile* in a Farm Environment Reveals the Presence of a New Toxigenic Strain Connected to Swine Production

**DOI:** 10.3389/fmicb.2022.858310

**Published:** 2022-04-14

**Authors:** Frederico Alves, Alexandra Nunes, Rita Castro, António Sequeira, Olga Moreira, Rui Matias, João Carlos Rodrigues, Leonor Silveira, João Paulo Gomes, Mónica Oleastro

**Affiliations:** ^1^Infectious Diseases Department, National Institute of Health Dr. Ricardo Jorge (INSA), Lisbon, Portugal; ^2^Faculty of Veterinary Medicine, Lusófona University, Lisbon, Portugal; ^3^CBIOS – Lusófona University Research Centre for Biosciences & Health Technologies, Lisbon, Portugal; ^4^National Zootechnical Station, National Institute for Agrarian and Veterinarian Research, Santarém, Portugal

**Keywords:** *Clostridioides difficile*, RT033, PaLoc, Pig, environment, One Health, transmission dynamics

## Abstract

The recent increase in community-acquired *Clostridioides difficile* infections discloses the shift in this bacterium epidemiology. This study aimed at establishing a transmission network involving One Health components, as well as assessing the zoonotic potential and genomic features of dominant clones. Samples were collected from different compartments of animal, human and environmental origin, from an animal production unit. *C. difficile* isolates were characterized for toxigenic profile by multiplex-PCR, while genetic diversity was evaluated by PCR-ribotyping and whole genome-based analysis. The overall *C. difficile* prevalence was 37.2% (70/188), and included samples from environmental (58.3%, 35/60) and animal (31.5%, 35/111) compartments; human samples (*n* = 17) taken from healthy workers were negative. A predominant clone from RT033 was found in almost 90% of the positive samples, including samples from all compartments connected to the pig production unit, with core-genome single nucleotide variant (SNV)-based Analysis supporting a clonal transmission between them (mean distance of 0.1 ± 0.1 core-SNVs). The isolates from this clone (herein designated PT RT033) were positive for all *C. difficile* toxin genes (*tcdA, tcdB, cdtA/cdtB*). The phyloGenetic positioning of this clone was clearly distinct from the classical RT033 cluster, suggesting a different evolutionary route. This new clone shares genomic features with several RTs from the clade 5 Sequence Type (ST) 11, including a complete pathogenicity locus (PaLoc) that is more similar to the one found in toxigenic strains and contrasting to the less virulent classical RT033 (*tcdA-, tcdB-, cdtA* + */cdtB* +). The presence of a *tcdA* gene truncated into two ORFs, not previously described, requires further evaluation concerning toxin functionality. We hypothesize that the unique combination of genetic elements found in the PT RT033 clone may contribute to host tropism and environmental dissemination and maintenance. This study constitutes the first report of a toxigenic RT033 clone and adds to the overall knowledge on Clade 5 sequence type 11, considered the *C. difficile* evolutionary lineage with the highest zoonotic potential. The presence of this clone in all compartments associated with the pig production unit suggests a transmission chain involving these animals and contributes to unveil the role played by animal and environmental reservoirs in this pathogen epidemiology.

## Introduction

*Clostridioides difficile* is a Gram-positive spore-forming anaerobic bacterium usually isolated from the feces of asymptomatic young animal and humans. The immature gut microbiota of these individuals allows the overgrowth of *C. difficile* over other enterobacterium. Overlooked as a major enteric pathogen for many years, a surge in diarrheic hospitalized patients was observed in recent years with the increase of antibiotic usage in healthcare facilities. *C. difficile* infection (CDI) severity can vary from mild diarrhea to life threatening pseudomembranous colitis, affecting mostly elderly hospitalized patients with a recent history of antimicrobial treatment. Nosocomial symptomatic infections were first associated with clindamycin consumption (Lance George et al., 1978). Other antibiotic groups that strongly associate with the development of symptomatic infection are second and third generation cephalosporins and fluoroquinolones (Brown et al., 2013; Slimings and Riley, 2014). The antibiotic-mediated alterations result in a gut microbiome derangement with a reduction of competitive flora that promote a shift in the gastrointestinal metabolic state to one that favors *C. difficile* germination, growth and toxin production (Giel et al., 2010; Buffie et al., 2012; Francis et al., 2013).

The *C. difficile* pathogenic potential is linked to the expression of two major cytotoxins encoded in the pathogenicity locus (PaLoc), the toxin A (TcdA) and the toxin B (TcdB; Voth and Ballard, 2005). Three supplementary regulatory genes are also present in the PaLoc: *tcdE*, *tcdR*, and *tcdC* (Chandrasekaran and Lacy, 2017). In addition, some strains of *C. difficile* produce a binary toxin (CDT) that contributes for the bacteria adhesion to the intestinal epithelial cells. Although its presence does not directly correlate to the severity of the clinical outcome (Berry et al., 2017), this toxin is present in the globally spread hypervirulent PCR ribotypes (RTs) 027 and 078.

The recent increase in community-acquired CDI affecting patients with no previous history of hospitalization discloses a shift in *C. difficile* epidemiology (Ofori et al., 2018) and unveils the urge to better understand the transmission dynamics taking place in the community, as well as identifying possible contamination sources and dissemination networks (De Roo and Regenbogen, 2020). Multiple studies have been published reporting the isolation of both toxigenic and non-toxigenic strains of *C. difficile*, similar to those found in humans. They include isolates from food-producing animal feces (Koene et al., 2012; Rodriguez et al., 2012, 2013; Schneeberg et al., 2013), from several foodstuff of animal origin, such as carcasses and abdominal viscera from abattoir samples (Rodriguez et al., 2013), retail meat products, seafood, fish and vegetable products (reviewed in Rodriguez-Palacios et al., 2020), and also from environmental samples (Janezic et al., 2016; Rodriguez et al., 2016). The extensive genetic overlap between strains was evidenced by the isolation of genetically related strains in animals and humans proving zoonotic transmission is possible (Knetsch et al., 2014, 2018; Werner et al., 2021). This is particularly notable in regard to the RT078, which is the most common RT isolated from pigs (Rodriguez et al., 2012; Stein et al., 2017), and also one of the most prevalent types found in hospitalized patients in Europe, with an increasing trend in prevalence (Bauer et al., 2011; Davies et al., 2016). In fact, whole-genome phylogenetic analysis reported by Knetsch et al. (2014) showing that pigs and farmers from the same farm were colonized with clonally related RT078 strains, support the possibility of interspecies transmission *via* fecal-oral route or a shared environmental source of infection. The RT078 belongs to clade 5, Multilocus Sequence Type (MLST) ST11, being highly divergent from other *C. difficile* clades, and comprising the RTs with the highest zoonotic potential which have recently emerged as common human pathogens (Knight et al., 2015). The close contact between humans and animals is enhanced in rural and farming environments, making it the ideal scenario to evaluate animal-human transmission.

Colonized animals and humans can shed oxygen resistant spores through the feces, allowing for an ubiquitarian presence of *C. difficile* in the environment. The ability of these spores to survive for a long period of time maintaining their infectious potential transforms contaminated soil and water into important reservoirs of public health concern (Kim et al., 1981; Al Saif and Brazier, 1996). The consumption of contaminated meat products, direct fecal-oral route (animal to person or person to person) and the ingestion of spores released into the environment are all possible transmission routes that need to be taken into account when assessing CDI risk factors (Janezic et al., 2016; Rodriguez Diaz et al., 2018). All these aspects make CDI a prime example of the interaction between animal, human and environmental health, disclosing the importance of the One Health approach when dealing with community circulating pathogens. Our research aimed at trying to establish a *C. difficile* transmission network involving all the One Health components using a pig farm as a proof-of-concept, as well as to assess the diversity of potential zoonotic types and to unveil genomic features of dominant clones.

## Materials and Methods

### Sampling Site Description and Sample Collection

**FIGURE 1:**
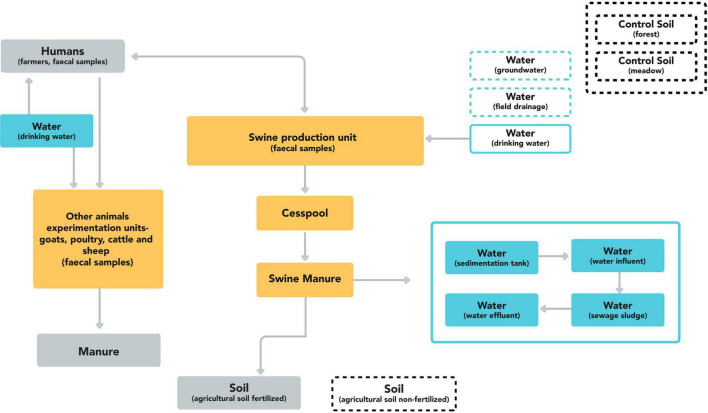
Schematic representation of the environmental, human, and animal compartments and the respective connection between them. The dashed line boxes indicate sampled compartments with no connection to the animal production units.

To evaluate the transmission dynamics of *C. difficile*, samples from multiple environmental, human and animal compartments from a single farm were studied. The sampling site, a zootechnical station located in Santarém, Portugal, was selected based on the possibility of assessing the interaction between all One Health components (Figure 1). The animal research nature of the chosen zootechnical station allowed to obtain biological material from production animals in conditions mimicking intensive animal farming. Multiple units from the 250 hectares were covered in the sampling process. Each unit was distant from the others and the animals were separated by species with no possibility of interspecies interaction or fecal contamination. From the pig production unit, both fattening and reproduction sows were included, as well as the piglets from different maternities of the reproduction unit. All the waste products from the pig production unit converged to the same collection area from where the cesspool samples were taken. The resulting waste was then subjected to solid and liquid phase separation. The manure derived from the separation process was then used for the agricultural soil fertilization. The liquid part was diverted to a wastewater treatment plant from which samples were also collected. After the appropriate treatment, the water was discharged into the river. The other group of manure samples comprised manure samples from cattle and sheep that were separately collected.

The sampling process took place between July 2020 and June 2021, at 2–3 time points. Environmental samples (*n* = 60) included agricultural soil samples, fertilized (*n* = 6) and non-fertilized (*n* = 9), non-agricultural soil, comprising the surrounding forest ground (*n* = 9) and the meadow (*n* = 9), swine manure (liquid and solid phases) (*n* = 6), pig production unit waste cesspools (*n* = 11) and manure from cattle (*n* = 2) and sheep (*n* = 1). The water samples were retrieved from the field drainage system, groundwater and drinking water, one of each (*n* = 3). Samples from the wastewater treatment plant (WTP) (*n* = 4) were also included, comprising one sample of each WTP influent, WTP effluent, WTP sedimentation tank and WTP sewage sludge. Animal fecal samples (*n* = 111) were taken from poultry (*n* = 9), sheep (*n* = 4), goats (*n* = 4), cattle (*n* = 8), and pigs, including fattening pigs (*n* = 18), reproduction sows (*n* = 25) and piglets (*n* = 43) from three different maternities of the reproduction unit. All animal stools were sampled from fresh droppings, except the piglets that were individually sampled by rectal swab. All samples were obtained with the farmers consent and collaboration following the animal wellbeing protocols in place in the animal production unit approved by the Portuguese Animal Welfare Authority (DGAV). Human fecal samples (*n* = 17) from healthy farmers and workers exposed to animal husbandry were also collected anonymously and voluntarily, the same individuals were sampled at two different times. None of the animals and humans included were showing gastrointestinal signs of disease at sampling time. Ethical approval for the study was obtained from the Health Ethics Commission from the National Institute of Health Dr Ricardo Jorge.

### Isolation of *Clostridioides difficile*

Regarding the fecal and manure samples, 0.5 g of each were enriched in 5 ml of *C. difficile* enrichment broth (proteose peptone 40.0 g/L, disodium hydrogen phosphate 5.0 g/L, potassium dihydrogen phosphate 1.0 g/L, magnesium sulfate 0.1 g/L, sodium chloride 2.0 g/L, fructose 6.0 g/L, sodium taurocholate 1.0 g/L, D-cycloserine 0.25 g/L, Cefoxitin 0.008 g/L) for a week, under anaerobic conditions generated using the anaerobic cultivation system Anoxomat (Anoxomat, Mart, Netherlands), at 37°C, changing the atmosphere every 48 h. This step was followed by ethanol shock (2.5 ml of the enrichment mixture in 2.5 ml of 96–100% ethanol for 1 h at room temperature) and centrifugation (2300 RCF for 10 min) before inoculation of the pellet onto ChromID^®^ *C. difficile* agar (bioMérieux, Marcy l’Etoile, France). The plates were incubated for 48 h under anaerobic conditions at 37°C. The rectal swabs were placed directly in 5 ml of *C. difficile* enrichment broth, and the following procedure was the same as described above.

The water samples were treated according to Janezic et al. (2016). Briefly, 50 ml of water was subjected to heat shock at 70°C for 20 min, followed by filtration through a 0.2 μm mixed cellulose ester membrane filter (Advantec^®^, California). Each filter was then enriched in 10 ml of *C. difficile* broth and incubated anaerobically at 37°C for 7 days, changing the atmosphere every 48 h. After enrichment, the filter was removed, and 5 ml of the mixture were centrifuged (2300 RCF for 10 min). The supernatant was removed and the pellet resuspended in 1 ml of 96–100% ethanol for 1 h before placing 2–3 drops on ChromID^®^ *C. difficile* agar and incubated anaerobically for 48 h.

The soil samples were treated according to Janezic et al. (2016) with slight modifications. The samples were broth enriched (25 g soil in 90 ml broth) for a week under the previously described conditions, followed by centrifugation of 50 ml of the suspension. The resulting supernatant was then subjected to heat shock at 70°C for 20 min and the entire volume was then filtered through a 0.2 μm mixed cellulose ester membrane filter (Advantec^®^). The filters were directly placed on ChromID^®^ *C. difficile* agar and incubated anaerobically at 37°C for 72 h. After incubation, between 2–5 presumptive colonies, were picked and cultured onto brucella blood agar with hemin and vitamin K1 (BBA) (BD BBL™, Heidelberg, Germany). The remaining colonies were swabbed from the filter, subjected to ethanol shock (1 ml 96100% ethanol for 1 h) and centrifuged (10500 RCF for 1 min). The pellet was then inoculated onto ChromID^®^ *C. difficile* agar and incubated anaerobically for 48 h.

### Toxins’ Profile, Ribotyping and Antimicrobial Susceptibility Testing

From the presumptively positive samples, based on colony morphologic characteristics, 3 to 5 colonies were picked, with exception of the piglets from which only 2 colonies were considered, and sub-cultured onto BBA under anaerobic conditions for 24 h at 37°C. Species confirmation was performed by MALDI-TOF (VITEK^®^ MS, bioMérieux). For each confirmed *C. difficile* positive colony, genomic DNA was extracted using the Isolate II Genomic DNA kit (Bioline, London, United Kingdom), according to manufacturer’s instructions. *C. difficile* isolates were first characterized by multiplex PCR, targeting *gluD* and the *tcdA*, *tcdB*, *cdtA* and *cdtB* toxin genes, according to Persson et al. (2009). Then, isolates were subjected to PCR-ribotyping using Bidet primers followed by capillary gel-based electrophoresis, according to Fawley et al. (2015). Antimicrobial susceptibility to moxifloxacin (5 μg), vancomycin (5 μg), metronidazole (5 μg) and rifampicin (30 μg) was performed by disk diffusion (Oxoid™, Hampshire, United Kingdom), and by Etest^®^ strips (bioMérieux) to clindamycin, using BBA and 24 h incubation under anaerobic conditions. *C. difficile* isolates were categorized as susceptible or resistant according to Erikstrup et al. (2012) for disk diffusion, and to the Clinical and Laboratory Standards Institute breakpoint for clindamycin (≥8 mg/L) [Clinical and Laboratory Standards Institute (CLSI), 2016].

### Whole Genome Sequencing

A total of 26 *C. difficile* RT033 isolates (here designated as PT RT033) were selected (see results for details; Supplementary Table 1) for WGS. In short, after quantitation using Qubit Fluorometer (Thermo Fisher Scientific, Porto Salvo), high-quality DNA samples were subjected to dual-indexed Nextera XT Illumina library preparation, cluster generation and paired-end short-read high throughput sequencing (2 × 150 bp) on an Illumina NextSeq550 equipment (Ilumina, San Diego) available at the National Institute of Health (INSA), according to the manufacturer’s instructions.

Reads quality control, species confirmation and bacterial *de novo* assembly were performed using the INNUca v4.2.2 pipeline^1^ (Llarena et al., 2018). Briefly, after reads’ quality analysis (FastQC v0.11.5^2^) and cleaning (Trimmomatic v0.38) (Bolger et al., 2014), genomes were assembled with SPAdes v3.14 (Bankevich et al., 2012) and subsequently improved using Pilon v1.23 (Walker et al., 2014). Species confirmation and contamination screening were assessed using Kraken v.2.0.7 (with 8GB database available at^3^) for both raw reads and final polished assemblies. MLST prediction was determined using mlst v2.18.1 software^4^. The 26 obtained draft genome sequences were annotated with RAST server v2.0^5^ (Aziz et al., 2008).

In order to understand the phylogenetic positioning of PT RT033 isolates, other clade 5 ST11 isolates (*n* = 57) (Knight et al., 2019) belonging to multiple RTs from environmental, animal and human sources from different geographic regions (*n* = 57) were also selected (Supplementary Table 1), and their paired reads were downloaded from ENA and *de novo* assembled as described above. The genetic relatedness among isolates was evaluated by a reference-based mapping strategy using Snippy v.4.5.1 software^6^. Quality improved reads of all 83 isolates were individually mapped against the reference *C. difficile* RT033 DSM 101085 strain (GenBank accession number: CP021219.1). Single-nucleotide variant (SNV) calling was performed on variant sites that filled the following criteria: (i) minimum mapping quality and minimum base quality of 20; (ii) minimum number of reads covering the variant position ≥10; and (iii) minimum proportion of 90% of reads differing from the reference. Core-SNVs were extracted using Snippy’s core module (snippy-core), by masking repetitive regions, mobile genetic elements (MGE) [like transposons and prohages (Riedel et al., 2020)] and recombinant regions (like both the PaLoc region and the S-layer cassette) of *C. difficile* DSM 101085 [encompassing a total of ∼6.5% (∼260 kb) of the genome], as their inclusion would bias the phylogeny. All reported SNVs/indels were visually inspected and carefully confirmed using the Integrative Genomics Viewer v2.11.0^7^ (Robinson et al., 2011). MEGA7 software^8^ (Kumar et al., 2016) was applied to calculate matrices of nucleotide distances and perform phylogenetic reconstructions over the obtained core-genome SNV alignment by using the Neighbor-Joining (NJ) method (Saitou and Nei, 1987) with the Maximum Composite Likelihood model to compute genetic distances (Tamura et al., 2004) and bootstrapping (1000 replicates) (Felsenstein, 1985).

### Extended Genetic Diversity Analysis

In order to identify virulence and putative antimicrobial resistance (AMR) genetic determinants, PT genome assemblies were queried against the following publicly available databases, using ABRIcate v1.0.0^9^ : CARD^10^, ResFinder^11^, ARG-ANNOT^12^ (Gupta et al., 2014), NCBI AMRFinderPlus^13^, MEGARES^14^, and VFDB^15^. The presence/absence and/or variability of each identified hit was confirmed by Snippy reference-based mapping, while BLASTp against the non-redundant (nr) protein sequences database was used to assess the potential protein function. The predicted AMR results were further compared with antimicrobial susceptibility testing results.

The genomic structure, organization and diversity of the PaLoc, binary toxin, *skin*^Cd^ element, S-layer cassette and accessory gene regulator (agr) regions on PT isolates was confirmed by Snippy reference-based mapping against *C. difficile* RT033 DSM 101085 or RT078 M120 (GenBank accession number: NC017174.1) strains and by RAST annotation. For comparative purposes, all the other clade 5 ST11 isolates (*n* = 57) were included in this analysis. Whenever it was possible, allele identification was conducted at the PubMLST platform (^16^ accessed in November 2021). Phylogeny of S-layer cassette was based on the concatenated sequences of *splA*, *secA*, *cwp2*, *lmbE*-like and *cwp66* genes, with NJ tree being generated using MEGA7 as described above.

As a means to potentiate isolate discrimination, assemblies of PT RT033 isolates were analysed using PHASTER web server^17^ (Arndt et al., 2016) to determine the presence of prophages. Only hits with intact phages were considered for further analysis, given that the detection of phage fragments could result from the assembly process hampering the distinction between presence of intact phages and phage remnants that were excised during the evolutionary process (for which the biological importance is even more uncertain). All identified hits were manually curated and when possible closely defined by predicting their attachment sites. The presence of plasmids was assessed through PlasmidFinder 2.1^18^, using default parameters with both trimmed reads and assemblies. All 83 ST-11 isolates were interrogated for the presence of *C. difficile* DSM 101085 plasmid (GenBank accession number: CP021320.1) by Snippy reference-based mapping.

The identification and structure of CRISPR-Cas systems (clustered regularly interspaced short palindromic repeats/CRISPR associated proteins) was predicted for all PT assembled genomes with CRISPRCasFinder^19^ web tool (accessed in September 2021). Only intact elements with the highest confidence score level were considered as legitimate hits. The occurrence and structure of each hit CRISPR-Cas system was confirmed by RAST annotation.

### Data Availability

Raw sequencing reads of all 26 *C. difficile* PT RT033 isolates used in the present study were deposited in the European Nucleotide Archive (ENA) under the BioProject accession number PRJEB49792.

## Results

### *Clostridioides difficile* Distribution and Characteristics by Compartment

A total of 188 samples were included in this study (Table 1). The overall *C. difficile* positivity rate was 37.2% (70/188), and included samples from environmental (58.3%, 35/60) and animal (31.5%, 35/111) compartments. No *C. difficile* was found in any of the 17 human samples analysed, taken from the same farmers in two distinct time points.

**TABLE 1:** Description of the samples analysed in the several compartments of the pig production unit and main findings.

Compartment	Sample type (n. of samples)	Sampling date	Prevalence (n. positive samples/N total)	Ribotype (n. isolates)	Toxigenic profile
Environment	Swine manure (*n* = 6)	July 2020–November 2020	66.7% (4/6)	RT033 (*n* = 8)	*tcdA^+^tcdB^+^cdt^+^*
	Manure (sheep and cattle) (*n* = 3)	November 2020	0% (0/3)	–	–
	Fertilized agricultural soil (*n* = 6)	December 2020	100% (6/6)	RT033 (*n* = 17)	*tcdA^+^tcdB^+^cdt^+^*
	Non-fertilized agricultural soil (*n* = 9)	December 2020	66.7% (6/9)	RT033 (*n* = 21)	*tcdA^+^tcdB^+^cdt^+^*
	Non-agricultural soil (Forest) (*n* = 9)	December 2020	22.2% (2/9)	RT033 (*n* = 2)	*tcdA^+^tcdB^+^cdt^+^*
				RT027 (*n* = 2)	*tcdA^+^tcdB^+^cdt^+^*
	Non-agricultural soil (Meadow) (*n* = 9)	December 2020	55.5% (5/9)	RT033 (*n* = 6)	*tcdA^+^tcdB^+^cdt^+^*
				RT720 (*n* = 2)	*tcdA^+^tcdB^+^cdt^–^*
				RT126 (*n* = 2)	*tcdA^+^tcdB^+^cdt^+^*
	Swine waste cesspools (*n* = 11)	December 2020	73% (8/11)	RT033 (*n* = 18)	*tcdA^+^tcdB^+^cdt^+^*
	Wastewater treatment plant (*n* = 4)	December 2020	100% (4/4)	RT033 (*n* = 7)	*tcdA^+^tcdB^+^cdt^+^*
				RT005 (*n* = 2)	*tcdA^+^tcdB^+^cdt^–^*
				RT071 (*n* = 2)	*tcdA^–^tcdB^–^cdt^–^*
				RT643 (*n* = 1)	*tcdA^+^tcdB^+^cdt^–^*
	Water (field drainage system, groundwater, drinking water) (*n* = 3)	July 2020	0% (0/3)	-	-
Animal	Poultry (*n* = 9)	November 2020	0% (0/9)	-	-
	Goats (*n* = 4)	November 2020	0% (0/4)	-	-
	Sheep (*n* = 4)	November 2020	25% (1/4)	RT126 (*n* = 2)	*tcdA^+^tcdB^+^cdt^+^*
	Cattle (*n* = 8)	November 2020	25% (2/8)	RT056 (*n* = 3)	*tcdA^+^tcdB^+^cdt^–^*
				RT643 (*n* = 1)	*tcdA^+^tcdB^+^cdt^–^*
				RT147 (*n* = 1)	*tcdA^+^tcdB^+^cdt^+^*
	Fattening pigs (*n* = 18)	December 2020	0% (0/18)	-	*-*
	Reproduction sows (*n* = 25)	December 2020–March 2021	24% (6/25)	RT033 (*n* = 16)	*tcdA^+^tcdB^+^cdt^+^*
	Piglets (*n* = 43)	March 2021	60.5% (26/43)	RT033 (*n* = 40)	*tcdA^+^tcdB^+^cdt^+^*
Human	Farmers* (*n* = 10)	February 2021	0% (0/10)	-	-
	Farmers* (*n* = 7)	June 2021	0% (0/10)	-	-

*Same individuals.

Regarding environmental samples, four out of six (66.7%) swine manure samples were positive for *C. difficile*, while the three manure samples from cattle and sheep were negative. All the six fertilized agricultural soil samples were positive for *C. difficile* and the positivity rate for the non-fertilized agricultural soil was 66.7% (6/9). Concerning the non-agriculture soil samples, *C. difficile* was found in 22.2% (2/9) and 55.5% (5/9) of the forest and meadow samples, respectively. From the pig’s waste cesspools, eight of 11 (73%) samples were positive for *C. difficile*. The four water samples from the WTP were positive for *C.diff*, contrasting with the three water samples collected from other sources that were all negative (Table 1).

Among the 35 positive samples from the environment, a total of 90 *C. difficile* isolates belonging to different toxigenic RTs were obtained (Table 1): eight isolates from swine manure (all RT033, *tcdA* +, *tcdB* +, *cdtA* + */cdtB* +); 17 from fertilized agricultural soil and 21 from non-fertilized agricultural soil (all RT033, *tcdA* +, *tcdB* +, *cdtA* + */cdtB* +); four isolates from the forest ground (two RT027, *tcdA* +, *tcdB* +, *cdtA* + */cdtB* +, two RT033, *tcdA* +, *tcdB* +, *cdtA* + */cdtB* +); 10 from the meadow (six RT033, *tcdA* +, *tcdB* +, *cdtA* + */cdtB* +, two RT720, *tcdA* +, *tcdB* +, *cdtA-/cdtB-*, and two RT126, *tcdA* +, *tcdB* +, *cdtA* + */cdtB* +); 18 from the swine cesspool (all RT033, *tcdA* +, *tcdB* +, *cdtA* + */cdtB* +); 12 isolates from the WTP (seven RT033, *tcdA* +, *tcdB* +, *cdtA* + */cdtB* +, two RT005, *tcdA* +, *tcdB* +, *cdtA-/cdtB-*, two RT071, *tcdA*-, *tcdB*-, *cdtA-/cdtB-*, and one belonging to RT643, *tcdA* +, *tcdB* +, *cdtA-/cdtB-*) (Table 1). Regarding antimicrobial resistance, only the two RT027 isolates from forest were resistant to moxifloxacin.

While none of the samples from poultry (*n* = 9) or goats (*n* = 4) revealed the presence of *C. difficile*, this pathogen was found in sheep (1/4, 25%) and cattle (2/8, 25%) samples. The swine population included in this study represented the majority of the animal samples collected, totalizing 86 out of 111 samples. The positivity rate of *C. difficile* in pigs was assessed separately according to category and age group. None of the 18 samples from fattening pigs was positive for *C. difficile*, contrasting to a positivity rate of 24% (6/25) and 60.5% (26/43) detected in the reproduction sows and in the piglets’ group, respectively. Regarding the piglet population, three maternity units were evaluated, in which the piglets were separated by age groups. Two litters from each maternity unit were sampled. The presence of *C. difficile* was associated with the piglet’s age, being more frequent in the ones aged between 1–2 weeks (12/14, 85.7%), followed by the 3-weeks old (9/14, 64.3%) and less frequent in the older ones with 1-month of age (5/15, 33.3%).

Among the 35 positive animal samples, a total of 63 *C. difficile* isolates belonging to different toxigenic RTs were obtained (Table 1): two from sheep (all RT126, *tcdA* +, *tcdB* +, *cdtA* + */cdtB* +), both presenting resistance to moxifloxacin; five from cattle (three RT056, *tcdA* +, *tcdB* +, *cdtA-/cdtB-*, one RT643, *tcdA* +, *tcdB* +, *cdtA-/cdtB*-, and one RT147, *tcdA* +, *tcdB* +, *cdtA* + */cdtB-*); 16 from the reproduction sows (all RT033, *tcdA* +, *tcdB* +, *cdtA* + */cdtB* +); 40 from piglets (all RT033, *tcdA* +, *tcdB* +, *cdtA* + */cdtB* +).

Overall, all compartments connected to the swine production unit (pigs, swine cesspool/manure, agricultural soil and WTP) presented the highest *C. difficile* positivity rate, and RT033 was the predominant type found in those compartments. All the RT033 isolates were susceptible to the five antibiotics tested.

### Genomic Characterization of the Predominant RT033

Considering the predominant distribution of RT033 (89.5%, 137/153) in all *C. difficile* positive compartments, a detailed genomic characterization of the isolates from this RT was undertaken. A group of 26 PT RT033 isolates representative of the different compartments (WTP, agricultural and control soils, manure, sows and piglets) was analysed (Supplementary Table 1).

**FIGURE 2:**
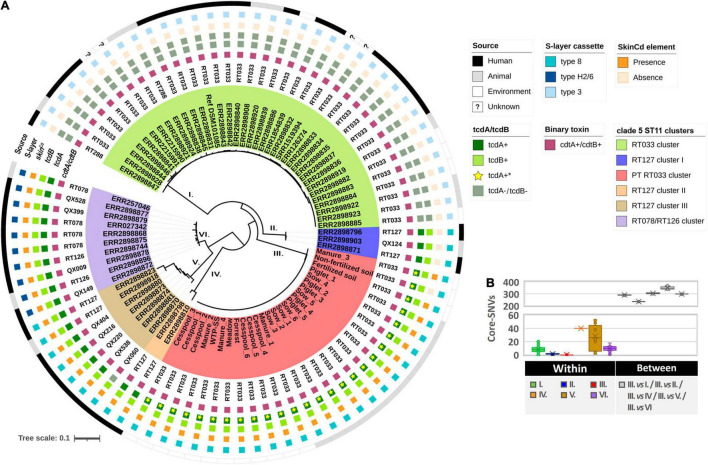
Phylogenetic positioning of PT RT033 clone within in the *Clostridioides difficile* clade 5 ST11 lineage. **(A)** The core-genome SNP-based phylogenetic tree was reconstructed using the Neighbor-Joining method (Saitou and Nei, 1987) with the Maximum Composite Likelihood model (Tamura et al., 2004). For all isolates (*n* = 84), the clade 5 ST11 sub-lineage (color-coded) is shown as well as the ribotype, the source, the S-layer cassette type, the skinCd element, the *tcdA*/*tcdB* and binary toxins. The tree was drawn using the iTOL website (https://itol.embl.de/) (Letunic and Bork, 2021). **(B)** Box Plots depicting the number of SNVs observed within and between all clade ST11 clusters. The horizontal line within each box marks the median, while the cross (“x”) represents the medium value.

As all PT RT033 isolates were expectably found to belong to clade 5 ST11, their phylogenetic positioning within the major RT sublineages (RT078, RT126, RT127, RT033, and RT288) of clade 5 ST11 (Knight et al., 2019) was first investigated. The core-genome SNV-based phylogenetic analysis shows a clear segregation of strains into distinct clusters (I-VI), with all PT RT033 isolates grouping together in an independent tree branch (III) (Figure 2A). Indeed, despite they were isolated from distinct compartments, PT RT033 isolates were found to be highly genetically related among them, differing only by a mean of 0.1 ± 0.1 core-SNVs. On the other hand, they exhibited a mean distance that ranged from 235.0 ± 13.1 to 346.8 ± 14.4 core-SNVs to clusters II (RT127 cluster I) and V (RT078/RT126 cluster), respectively (Figure 2B). Surprisingly, when compared to the classical RT033 cluster (I), whose isolates displayed a mean of 8.0 ± 1.2 core-SNVs among them, the PT RT033 cluster distant a mean of 290.5 ± 14.1 core-SNVs. Overall, these results point for a clonal origin of PT RT033 isolates, clearly distinct from the remaining clade 5 ST11 sublineages, including the classical RT033 cluster.

**FIGURE 3:**
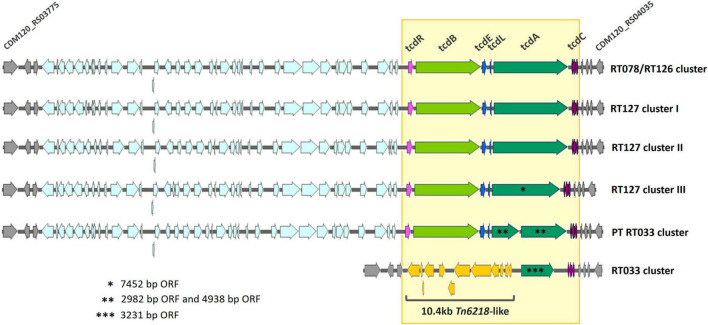
PaLoc genetic diversity within the *Clostridioides difficile* clade 5 ST11 lineage. For all clusters, ORFs orientation is depicted by arrows, while PaLoc region is highlighted by a yellow box with *tcdR*, *tcdB, tcdE*, *tcdL* and *tcdA* genes differently colored. The Tn6218-like element, exclusively found in the classic RT033 cluster, is shown in yellow arrows. Flanking genes (homologous to all clusters) of the PaLoc-containing region are represented by gray arrows, where extremes designated according to RT078 reference M120 genome (NC017174.1).

A deeper genomic inspection of the PT RT033 cluster allowed to pinpoint several genomic features that reinforce the distance observed to the classical RT033 cluster. One of the major discrepancies was found in the PaLoc (Figure 3) since RT033 has been traditionally considered as non-toxigenic due to the insertion of a 10.4 kb Tn6218-like element that has resulted in a deletion of most genes involved in toxins’ production, regulation and secretion facilitation, leaving only a truncated *tcdA* pseudogene and a disrupted *tcdC* (Elliott et al., 2014). In contrast, the PT RT033 clone exhibit a large genomic region (∼58 kb) that include an 18.5 kb complete PaLoc as well as its immediately upstream region, with a genetic organization similar to the remaining clade 5 ST11 sublineages. Indeed, all *tcdR*, *tcdB*, *tcdE*, *tcdL*, and *tcdA* PaLoc genes, as well as the disrupted *tcdC* typically present in clade 5 sublineages, were found in PT RT033. However, some discrepancies were observed that make cluster’s PT RT033 PaLoc unique. Indeed, when compared with RT078 reference M120 strain (NC017174.1), both *tcdR* and *tcdB* displayed a missense substitution (533A > G| Tyr178Cys and 6658G > A| Asp22220Asn, respectively), while *tcdA* is truncated in two smaller ORFs of 2982 bp and 4938 bp due to a C→T alteration at position 2980, resulting in a prematurely gained stop codon (at 994aa). Moreover, three additional missense alterations (5333T > A| Met1778Lys; 5710A > G| Arg1901Gly and 5997T > C| Asp1999Asp) were found in the major *tcdA* ORF (Figure 3).

Likewise, disparities were also found regarding the binary toxin, with the PT RT033 clone harboring a novel *cdtA* synonymous variant (936T > G) and a wild-type *cdtB* gene (allele 21) identical to all ST11 toxigenic clusters (except for RT127 cluster II, allele 48), differing from the classical RT033 *cdtB* by a synonymous substitution (allele 14). Regarding *cdtR*, PT RT033 isolates were found to harbor the wild-type 747 bp allele characteristically exhibited by non-RT078/RT126 strains.

The S-layer cassette diversity was also evaluated through phylogenetic characterization of concatenated *slpA*, *secA*, *cwp2*, *lmbE*-like and *cwp66* gene sequences (totalizing ∼10 kb) (Supplementary Figure 1), as they were shown to be the most polymorphic within this region (Dingle et al., 2013). All PT RT033 isolates were clearly segregated within the RT127 cluster branch tree, for which S-layer cassette was classified as type 8 (Knight et al., 2019). Interestingly, they are distanced by 1807.8 ± 29.1 and 1342.6 ± 31.6 nucleotides from the classical RT033 cluster (S-layer type 3) and RT078/RT126 cluster (S-layer type H2/6), respectively.

**FIGURE 4:**
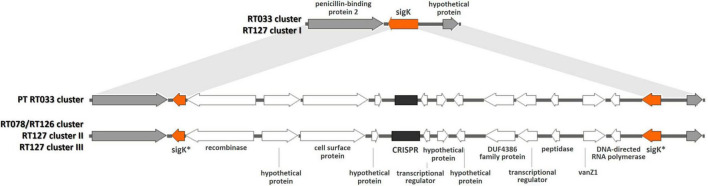
Genetic structure of the *skin^Cd^* element of *Clostridioides difficile* PT RT033 clone. For all clade 5 ST11 clusters, ORFs orientation is depicted by arrows. Intact or interrupted (*) *sigK* genes are represented by orange arrows, while homologous flanking genes are shown in gray arrows. All 12 ORFs that compose *skin^Cd^* element are represented by white arrows, and CRISPR element is depicted as a black box.

Moreover, in PT RT033 isolates, the sporulation-specific gene *sigK* is interrupted by a 10 kb phage-like *skin*^Cd^ element with similar gene content and structure to that seen in RT078/RT126 cluster and RT127 clusters II and III (Figure 4). This element is composed of twelve ORFs, including its site-specific recombinase [required for excision in the mother cell during sporulation (Haraldsen and Sonenshein, 2003)] and of the *vanZ1* gene [shown to confer low-level of resistance to the glycopeptide antibiotic teicoplanin (Woods et al., 2018)], as well as a CRISPR element without *cas*-associated genes. The only exception occurred for the PT RT033 non-fertilized soil isolate that possesses an intact *sigk* like both the classical RT033 cluster and the RT127 cluster I.

Regarding the presence of other MGEs, no plasmid was found in any PT RT033 draft genomes, including that present in the reference RT033 DSM101085 strain (CP021320.1). On the other hand, genome inspection of all 26 PT RT033 isolates revealed the presence of prophage regions with homology to four known *Clostridium* phages. Similarly to the reference RT033 DSM101085 strain (NZ_CP021329.1), all PT RT033 isolates were found to carry two distinct intact prophages, namely: (*i*) an ∼48–58 kb phiCDHM19-like prophage containing 76–83 ORFs and a predicted CRISPR array without *cas* genes, and (*ii*) an ∼46 kb phiMM03-like or phiMMP01-like prophage with 76 ORFs. The only exception was the Manure_3 isolate, for which a phiCP340-like prophage was additionally identified. However, no association was found between the presence or combination of phages and the different compartments from which samples were collected. There is, however, a difference concerning the insertion sites of the phiCDHM19-like prophage, which is inserted between ORFs CDIF101085_01985 (c-di-GMP-I riboswitch) and CDIF101085_02107 (hypothetical protein) for reference RT033, whereas it interrupts the homolog CDIF101085_00958 (∼1143 bp) that codes for an aldo/keto reductase in PT isolates, resulting in a smaller ORF (∼552 bp). On the other hand, PT RT033 isolates share the same insertion site as reference RT033 for phiMM03-like prophage, which disrupts an ABC transporter coding gene (CDIF101085_03033). The only exception occurred for Manure_2 isolate, in which a phiCDHM19-like prophage was found at this location instead of a phiMM03-like prophage (i.e., interrupting the CDIF101085_03033 homolog). Interestingly, both phiMM03- and phiMM01-like prophages were found to carry an additional *agr1-*like locus containing only *agrB* and *agrD* genes, which contrasts to the *agr3* locus displayed by the classical RT033 cluster that also contains the *agrC* gene in phiMM03 phage (Riedel et al., 2020).

Finally, no AMR genetic determinants were found *in silico* for the 26 PT RT033 isolates, supporting and complementing the obtained phenotypic results.

## Discussion

This study aimed firstly at evaluating the transmission dynamics of *C. difficile* focusing on the role played by environmental reservoirs, using a model centered on a pig farm. The overall positivity rate of 37.2% observed in this study is in agreement with other studies conducted in swine farms (Andrés-Lasheras et al., 2017; Krutova et al., 2018) and suggests a key role of animal production units in the CDI panorama, acting as possible reservoirs of toxigenic strains. The prevalence of *C. difficile* was found to be almost 2-fold higher in environmental samples (58.3%) than in animal samples (31.5%). The fact that most environmental samples included in this study were related to the pig production units may have accounted for the prevalence noted, since the isolation rate was higher in pigs, especially in piglets, than in other species, suggesting a transmission cycle involving these animals. The role of the environment in the transmission chain has been reported before (Al Saif and Brazier, 1996; Janezic et al., 2016), particularly the piggery enclosure with a study by O’Shaughnessy et al. (2019) also reporting a prevalence above 50%. Worth noting is the fact that all samples from the WTP and fertilized agricultural soil (a total of 10 samples), which were directly connected to the swine manure, were positive for *C. difficile*. This animal production by-product is integrated back into the environment, used for agricultural soil fertilization or released into water bodies, contributing to the environmental dissemination of toxigenic strains (Al Saif and Brazier, 1996; Le Maréchal et al., 2020; Lim et al., 2020).

Regarding animal samples, the positivity rate was around 25% in cattle, sheep and pigs, the exception being the piglet group from which 60.5% were positive. This higher prevalence might be attributed to microbiota immaturity which facilitates *C. difficile* overgrowth and isolation (Grześkowiak et al., 2019). When looking into the piglet population it is notable that the prevalence decreased with age, with a positivity rate of 85.7% in the 1–2 weeks old group, 64.3% at 3-weeks old and decreasing to 33.3% in 1 month old animals. Together with previous studies (Weese et al., 2010; Susick et al., 2012), our results corroborate the importance of intestinal flora maturity in CDI control. Considering the high prevalence of *C. difficile* found in the environment, it is likely that piglet colonization arises from suckling on contaminated teats or from direct fecal-oral route (Hopman et al., 2011).

Even though the humans were selected based on animal proximity, none of their fecal samples was positive for *C. difficile.* The fact that these were all healthy adults with no history of recent antimicrobial administration may have accounted for the negative results. It is also possible that the handling and hygiene practices adopted in the zootechnical station are effective in preventing workers from becoming colonized by the circulating pathogens. Nevertheless, the high prevalence of *C. difficle* isolated from the environment reveals the importance it may hold as a plausible reservoir of toxigenic strains that can potentially be the source of human community acquired CDI.

Despite the clear predominance of RT033, showing a prevalence of 89.5%, a high diversity of toxigenic RTs was found. The inclusion of different animal species as well as different environmental compartments justifies the diversity found and is supported by other farm related studies (Andrés-Lasheras et al., 2017; Krijger et al., 2019). Some of the remaining isolates observed in this study also belonged to RTs with zoonotic potential for which symptomatic infections are being increasingly reported in hospitalized patients, such as the case of RT126 [(Davies et al., 2016) and data from the laboratory-based epidemiological surveillance of CDI in Portugal, Portuguese National Institute of Health]. The resistance to moxifloxacin found in RT027 from the forest soil and RT126 from a sheep deserves special attention since fluoroquinolone resistance is mainly found in epidemic strains, particularly RT027 responsible for several outbreaks of nosocomial nature (Clements et al., 2010).

The PT RT033 isolates were found in all compartments connected to the pig production unit. The high genetic relatedness found among isolates (0.1 ± 0.1 core-SNVs) (Figure 2B) supports a clonal transmission between animal and environmental compartments, as a threshold of ≤2 SNV in the core genome is usually accepted to define clonally related strains (Bletz et al., 2018). Considering the almost ubiquitous presence of the isolated clone in the environmental compartments, especially in the ones linked to the swine production unit, the introduction of new animals that rapidly become colonized and continue to contribute to spore dissemination, allows for the maintenance of the farm transmission dynamic.

Most notably, the PT RT033 clone was clustered separately from the previously described RT033 highly suggesting a different evolutionary path (Figure 2A). One of the most significant disparities was found for the PaLoc with the PT RT033 clone displaying a complete locus similar to that harbored by other clade 5 ST11 toxigenic strains (Figure 3), where all the toxin and regulatory genes (*tcdR, tcdB, tcdE, tcdL*, and *tcdA*) are present, including an aberrant *tcdC* typical of some clade 5 sublineages. Nevertheless, some unique features like the truncation of the *tcdA* gene into two smaller ORFs and a few missense nucleotide substitutions, make this a distinctive PaLoc genotype not previously described. To our knowledge this is the first report of a toxigenic RT033 strain, contributing to the overall PaLoc diversity. The PaLoc loss in the classical RT033 is considered a fairly recent evolutionary event resulting from the acquisition of the Tn6218 mobile element by a previously toxigenic ancestral (Elliott et al., 2014). Altogether, considering that PT RT033 harbors a complete PaLoc and it belongs to a distinct genetic cluster, these findings suggest that it might be a more ancestral RT033 clone that underwent a separate evolution route which did not include the acquisition of the mobile element responsible for the typical deletion in the PaLoc.

Further phenotypical characterization is needed to evaluate Toxin A expression and cytotoxic effect. The role played by the binary toxin in CDI pathogenesis is still under debate but it is generally assumed as a virulence increasing factor that works synergistically with toxins A and B contributing to poorer prognosis (Gerding et al., 2013). Nonetheless, strains that exclusively produce the binary toxin are still considered as disease-causing agents (Riedel et al., 2020). Therefore, it is highly expected that the PT RT033 clone holds an increased pathogenic potential as compared the classic RT033 strains.

Regarding the S-layer cassette composition, all PT RT033 isolates, except one, were classified as having a type 8 S-layer similar to the one found in the RT127 cluster. These genetic elements behave independently from the genome and suffer a much higher recombination rate *via* homologous recombination. This phenomenon of S-layer cassette switching, occurring independently from the strains genotype, contrasts with the low mutation rate found within each cassette (Supplementary Figure 1), with very little diversity being reported between isolates with the same variant (Dingle et al., 2013). It is possible that PT RT033 clone acquired its S-layer cassette from other RT before establishing its dominance in the farm, decreasing the chances of further recombination events that would result in a higher cassette diversity. Considering the cell surface is closely related to the bacterial antigenic properties it is likely that the PT RT033 triggers a different antigenic response than the classic RT033 (Dingle et al., 2013). Given the role of the outer membrane in the host-pathogen interaction (Steinberg and Snitkin, 2020) it can also be speculated that the PT RT033 clone, having a S-layer cassette closer to the ones found in RTs more commonly found in pigs (e.g., RT078/RT126), may hold a similar host tropism.

*Clostridioides difficile* spore formation is a complex process that depends on many regulatory genes, one of which is the *sigK* gene. This sigma factor plays an essential role in the late stage of the spore formation chain of events, being responsible for endospore coat and exospore synthesis as well as being indispensable to the release of spores into the environment (Saujet et al., 2014). Unlike the classic RT033 strains, the presently described PT RT033 clone harbors a phage-like *skin*^Cd^ element that interrupts the *sigK* gene (Figure 4). The *skin*^Cd^ excision is needed for *sigK* and late sporulation related genes to be expressed. Previous studies suggest that *skin*^Cd^– and *skin*^Cd^ + *C. difficile* strains display different sporulation properties, the latter presenting an increased sporulation efficiency (Haraldsen and Sonenshein, 2003). The presence of this phage-like element in the PT RT033 isolates may translate into an increased sporulation fitness when compared to the typical RT033, which can be partially responsible for the maintenance of the transmission network observed in the farm.

Regarding other MGE, the presence of phiMM01 phage in PT RT033 is noteworthy since it has not been described to date in this RT, with previous studies reporting its presence in other ST11 RTs (e.g., RT126 and RT127) (Knight et al., 2019). *C. difficile* prophages have been proven to influence gene expression, including virulence factors and antimicrobial resistance genes transduction (Brouwer et al., 2013; Wasels et al., 2014), and a more in-depth analysis of the genetic influence this phage may have in the newly described clone is worth exploring. In addition, PT RT033 shares the phiCDHM19 and phiMM03 prophages with the classical RT033, although the insertion site for the phiCDHM19 prophage is different, interrupting an aldo/keto reductase gene. This protein belongs to a superfamily of enzymes which functions are related to the reduction of aldehydes and ketones. Microorganisms capable of producing these enzymes hold a selective advantage, as these are mutagenic compounds that can be found in the environment and can represent a danger to microbial cells. Although it is important to explore if the insertion of a prophage in this ORF has any influence in AKR gene expression, it is plausible that, even if this ORF becomes dysfunctional by such event, other enzymes partially support the reaction resulting in no significant disadvantage for the cell (Ellis, 2002).

Regarding *agr* loci, the classical RT033 isolates harbor three *agr* loci, the typical *agr1*, one *agr1*-like, and an *agr3*, the last one located in phiMMP03 prophage (Riedel et al., 2020). However, the PT RT033 isolates, besides the two *agr1* loci, harbors a third *agr1*-like locus located in either phiMM03 or phiMM01 prophages. To our knowledge, this is the first report of an *agr1*-like locus in an MGE. Considering the role *agr* genes pose in contributing to the virulence and colonization factors by quorum sensing regulation (Darkoh et al., 2017), the presence of such element in a prophage that can be subjected to horizontal gene transfer, may influence the behavior of the bacterial community considered in this study (Hargreaves et al., 2014; Okada et al., 2020). Further studies are still needed to uncover the role of this third *agr1*-like locus in the PT RT033 clone, but it is possible that it may influence biofilm production through quorum sensing regulation contributing to bacterial persistence in the environment, thus allowing for the maintenance of the transmission cycle.

Finally, no AMR determinants were found in any of the PT RT033 isolates, which may be due to the experimental nature of the zootechnical station, where animal exposure to antibiotics is much lower than in intensive animal farming. The low antimicrobial pressure may have allowed the clone to establish well in the ecosystem, not being overthrown by more resistance strains that would be expected to thrive in a more antibiotic saturated environment.

## Conclusion

The present study comes as a valuable contribution to the overall knowledge on the diversity of the clade 5 ST11, shedding light on the evolutionary path, not only of this clade but also of the *C. difficile* as a species. The PT RT033 shows a unique combination of genetic features found in other clade 5 RTs but never before reported in RT033. Some of the newly described characteristics are closely related with sporulation properties, biofilm production and outer membrane properties, and could possibly be accountable for the generalized spread and environmental maintenance of this clone in the studied animal production unit. Considering this is the first report of a toxigenic RT033 strain, its pathogenic potential cannot be ignored. Still, further studies are still needed in order to understand the functionality of the PT RT033 clone PaLoc and its zoonotic potential. Given the close animal-human interaction taking place in animal production units, an interspecies leap can occur and human infections with circulating clones may arise as a consequence of environmental or fecal-oral contamination.

## Data Availability Statement

The datasets presented in this study can be found in online repositories. The names of the repository/repositories and accession number(s) can be found in the article/Supplementary Material.

## Ethics Statement

The studies involving human participants were reviewed and approved by Comissão de Ética do Instituto Nacional de Saúde Doutor Ricardo Jorge, I. P. The patients/participants provided their written informed consent to participate in this study.

## Author Contributions

FA, RC, and MO conceptualized the study. FA, AN, RC, AS, OM, RM, JR, LS, JG, and MO contributed to the methodology. FA, AN, and MO worked on the original draft of the manuscript. FA, AN, LS, JG, and MO reviewed and edited the manuscript. All authors have read and agreed to the published version of the manuscript.

## Conflict of Interest

The authors declare that the research was conducted in the absence of any commercial or financial relationships that could be construed as a potential conflict of interest.

## Publisher’s Note

All claims expressed in this article are solely those of the authors and do not necessarily represent those of their affiliated organizations, or those of the publisher, the editors and the reviewers. Any product that may be evaluated in this article, or claim that may be made by its manufacturer, is not guaranteed or endorsed by the publisher.

## Footnotes

1
https://github.com/B-UMMI/INNUca

2
http://www.bioinformatics.babraham.ac.uk/projects/fastqc/

3
https://ccb.jhu.edu/software/kraken/

4
https://github.com/tseemann/mlst

5
http://rast.nmpdr.org/

6
https://github.com/tseemann/snippy

7
http://software.broadinstitute.org/software/igv/

8
http://www.megasoftware.net

9
https://github.com/tseemann/abricate

10
https://card.mcmaster.ca/

11
https://cge.cbs.dtu.dk/services/ResFinder/

12
https://www.mediterranee-infection.com/acces-ressources/base-de-donnees/arg-annot-2/

13
http://www.ncbi.nlm.nih.gov/pathogens/antimicrobial-resistance/AMRFinder/

14
https://megares.meglab.org/

15
http://www.mgc.ac.cn/VFs/

16
http://pubmlst.org/

17
https://phaster.ca/

18
http://cge.cbs.dtu.dk/services/PlasmidFinder/

19
http://crisprcas.i2bc.paris-saclay.fr/
