# Supplementary figures and images for: Assessment of the Transmission Dynamics of Clostridioides difficile in a Farm Environment Reveals the Presence of a New Toxigenic Strain Connected to Swine Production
Source: Front Microbiol. 2022 Apr 14;13:858310. doi: 10.3389/fmicb.2022.858310 (PMC9050547; doi:10.3389/fmicb.2022.858310)

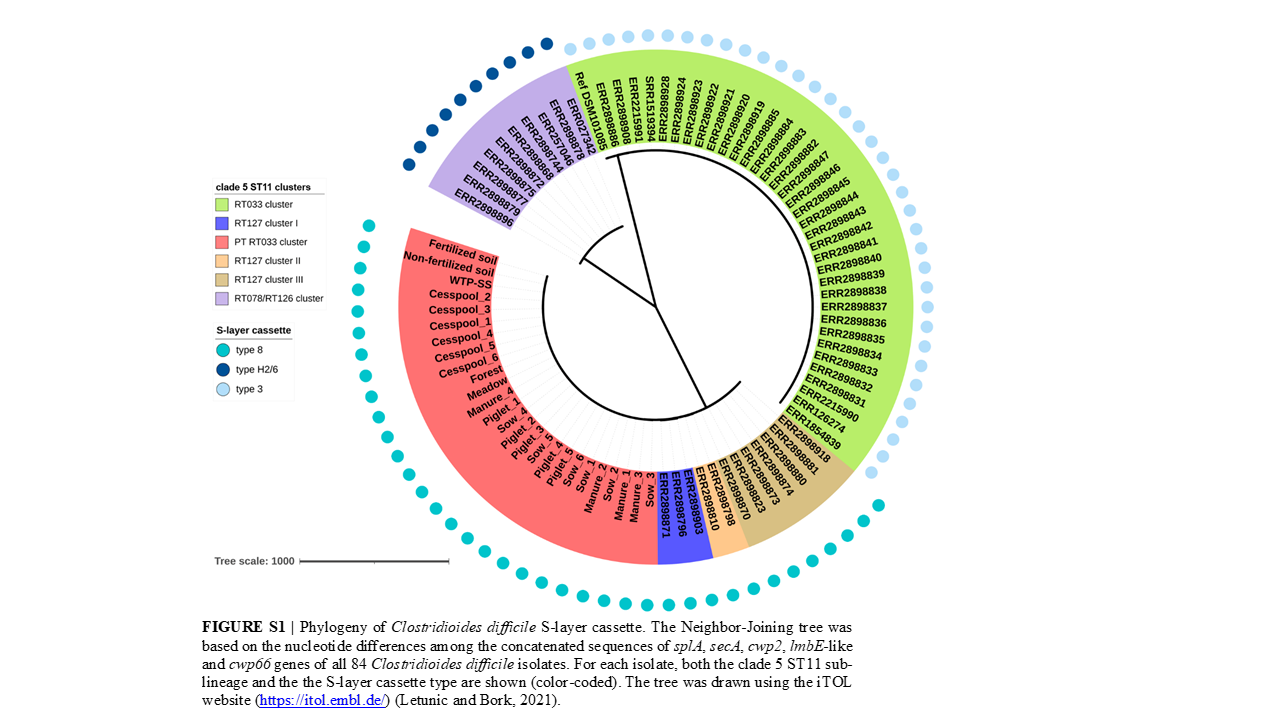

Supplement: Supplementary file 1 [file Image_1.TIF]
